# Association of Systolic Blood Pressure and Cerebral Collateral Flow in Acute Ischemic Stroke by Stroke Subtype

**DOI:** 10.3389/fneur.2022.863483

**Published:** 2022-05-13

**Authors:** Jae Eun Sim, Jong-Won Chung, Woo-Keun Seo, Oh Young Bang, Gyeong-Moon Kim

**Affiliations:** Department of Neurology, Samsung Medical Center, Sungkyunkwan University School of Medicine, Seoul, South Korea

**Keywords:** ischemic stroke, atherothrombotic stroke, cardiac emboli, collateral circulation, middle cerebral artery occlusion

## Abstract

**Background and Purpose:**

Collateral flow in acute ischemic stroke is known as a predictor of treatment outcome and long-term prognosis. However, factors determining the initial collateral flow remain unclear. We investigated factors related to collateral flow in patients with acute ischemic stroke caused by large vessel occlusion (AIS-LVO) and further analyzed the results according to stroke etiology.

**Methods:**

This was a retrospective study using prospective stroke registry data from a single university hospital from October 2014 to May 2021. AIS-LVO with middle cerebral artery M1 occlusion identified by pre-treatment multiphasic computed tomography angiography was included. Collateral flow score was graded on a 6-point ordinal scale according to pial arterial filling.

**Results:**

A total of 74 patients [cardioembolism (CE): 57; large artery atherosclerosis (LAA): 17] was included. The mean age of all patients was 72.2 ± 11.7 years, and 37.8 % (*n* = 28) were men. Multivariate regression analysis showed that initial SBP [odds ratio (OR): 0.994; 95% confidence interval (*CI*): 0.990–0.998; *p* = 0.002] and stroke etiology (*OR*: 0.718; 95% *CI*: 0.548–0.940; *p* = 0.019) were independent factors of the collateral flow grade. Collateral flow grade was independently associated with initial SBP in the CE group (*OR*: 0.993; 95% *CI*: 0.989–0.998; *p* = 0.004) but not in the LAA group (*OR*: 0.992; 95% *CI*: 0.980–1.004; *p* = 0.218). Initial SBP was significantly correlated with NIHSS score in the CE group but not in the LAA group (*r*^2^= 0.091, *p* = 0.023; *r*^2^ = 0.043, *p* = 0.426, respectively).

**Conclusions:**

Elevated initial SBP was associated with poor cerebral collateral flow and more severe symptoms in the CE group, but not in the LAA group in patients with AIS-LVO. These findings suggest differential effects of initial SBP elevation on collateral flow by stroke subtypes.

## Introduction

Collateral flow is an important feature of cerebral circulation when major arteries are occluded, which varies depending on individuals. Collateral flow in acute ischemic stroke is known as a predictor of acute thrombolytic and endovascular therapy outcome as well as long-term prognosis (1–5). Patients with a cervical large artery atherosclerosis (LAA) have a more extensive cerebral collateral circulation and a better functional outcome at 90 days than those with a cardioembolic (CE) stroke (6). Previous studies have also suggested that LAA stroke is associated with better collateral flow than CE stroke (6–9). Theoretically, LAA in humans develops over decades, which might promote the gradual development of cerebral collateral flow. For example, patients with a severe (71–99%) stenosis have a better collateral flow than those with a moderate (51–70%) stenosis (6). In contrast, since CE stroke is not accompanied by chronic cerebral hypoperfusion, the chances of collateral artery formation and recruitment are less likely to occur in these patients.

Although the importance of collateral flow has attracted more attention over the past decade, studies on the factors which determine collateral flow are not well understood. The potential relationship of collateral flow with history of cardiovascular risk factors, such as hypertension, congestive heart failure, hyperlipidemia, and diabetes might exhibit complex interactions. Calleja et al. have reported that diabetes is significantly associated with poorer collateral flow on admission (1).A history of hypertension has also been more frequently found among patients with poor collateral flow (2, 10, 11). Menon et al. have found that metabolic syndrome, hyperuricemia, and age are associated with poor collateral flow in patients with acute ischemic stroke (12). In case of coronary collateral flow, eGFR is an independent affecting factor (13).

We hypothesize that factors related to collateral flow in patients with acute ischemic stroke caused by large vessel occlusion (AIS-LVO) are different according to stroke etiology. In this study, we investigated the extensive physiologic, laboratory, and imaging parameters which have been reported to be possibly related with collateral flow in patients with AIS-LVO. We further analyzed the results according to stroke etiology with the assumption that stroke subtype may influence the collateral development.

## Materials and Methods

### Study Design

This was a retrospective study using prospective stroke registry data from a single university hospital (Samsung Medical Center, Seoul, South Korea). All patients underwent a standard unenhanced CT (5-mm section thickness), followed by a head and neck multiphasic computed tomography angiography (mCTA). In addition, both baseline National Institutes of Health Stroke Scale (NIHSS) score and Alberta stroke program early CT (ASPECT) score were assessed. Each patient (or their guardian) gave written informed consent for participation in this study. The complete study protocol was approved by the Ethical Committee of Samsung Seoul Medical Center.

### Patient Selection

A flowchart of patient selection is shown in Supplementary Figure 1. Inclusion criteria were: (1) patients who were admitted within 7 days of symptom onset with middle cerebral artery (MCA) M1 occlusion from October 2014 to May 2021; and (2) those with pre-treatment mCTA evaluation. A total of 3,187 patients with AIS who had mCTA were reviewed from our database. Patients other than MCA stroke (*n* = 1,709), without LVO (*n* = 1,357), with other etiologies or undeterminedetiology were successively excluded. Finally, 74 eligible patients were identified for analysis.

We comprehensively collected demographic data and clinical information, laboratory findings, and vascular risk factors. Initial systolic blood pressure (SBP) was recorded on arrival at the emergency department or after onset of symptoms in hospital stroke cases. Based on comprehensive data and results of workups, stroke pathogenesis was determined according to the Stop Stroke Study TOAST classification (14). Smoking habit was categorized as current smoker or ex-smoker/never smoker. Hypertension was defined as previous diagnosis of hypertension (>140/90 mmHg) or use of antihypertensive treatment for blood pressure control. Diabetes was defined as previous diagnosis of diabetes or current use of antidiabetic drugs. Hypercholesterolemia was defined as previous diagnosis of hypercholesterolemia or current use of antihyperlipidemic medications.

### Multiphasic Computed Tomography Angiography

The mCTA collateral score is a simple scoring system that allows quick evaluation of collateral vessel filling delay in AIS. All post processing of mCTA was automated and available for review within 2–3 min of CTA. The mCTA provides three time-resolved images of pial arterial filling in the whole brain, unlike conventional single-phase CTA. The pial arterial filling in the ischemic territory was measured in the first phase of CTA and during mCTA by comparing it to similar arteries in the unaffected hemisphere using a 6-point scale (15). A score on a scale of 0–5 was given, with 0 being the worst and 5 being the best: (**0**, no vessels visible in the affected hemisphere in any phase; **1**, only a few vessels were visible in the affected hemisphere in any phase; **2**, a filling delay of two phases in the affected hemisphere with a significantly reduced number of vessels in the ischemic territory, or one phase delay showing regions without visible vessels; **3**, a filling delay of two phases in the affected hemisphere, or a delay of one phase with a significantly reduced number of vessels in the ischemic territory; **4**, a filling delay of one phase in the affected hemisphere, but the extent and prominence of pial vessels were the same; **5**, no filling delay compared to the asymptomatic contralateral hemisphere, with normal pial vessels in the affected hemisphere). We showed the example of mCTA score in Supplementary Figure 2. We dichotomized scores of 0–3 and 4–5 to be “poor” and “good” collateral flow, respectively (15).

### Statistical Analysis

Initially, we divided two groups by stroke etiology. Nominal variables are expressed as counts and percentages. Continuous values are presented as mean ± standard deviation (SD). Continuous variables were analyzed with Student's *t*-test and Wilcoxon rank sum test. Categorical variables were analyzed with χ^2^-test and Fisher's exact test. Binary logistic regression analyses using these outcome measures as dependent variables were repeated for different variables of interest. After we found that initial systolic SBP was related to collateral grade, we performed appropriate multivariable binary logistic regression analyses to obtain odds ratio. Adjustments for covariates were performed for anterior cerebral artery (ACA) A1 agenesis and fetal posterior cerebral artery (PCA), which were variables showing differences in the univariable analysis (*p* < 0.1). Further adjustment covariates were performed for age and ASPECT, which were variables may influence leptomeningeal collaterals and the peripheral micro-perfusion. Subsequently, adjustment was performed for initial SBP. R software (R version 4.1.0, The R Foundation for Statistical Computing) was used for all statistical analysis. 2-tailed *p*-values <0.05 were considered statistically significant.

## Results

### Baseline Characteristics

**Figure 1:**
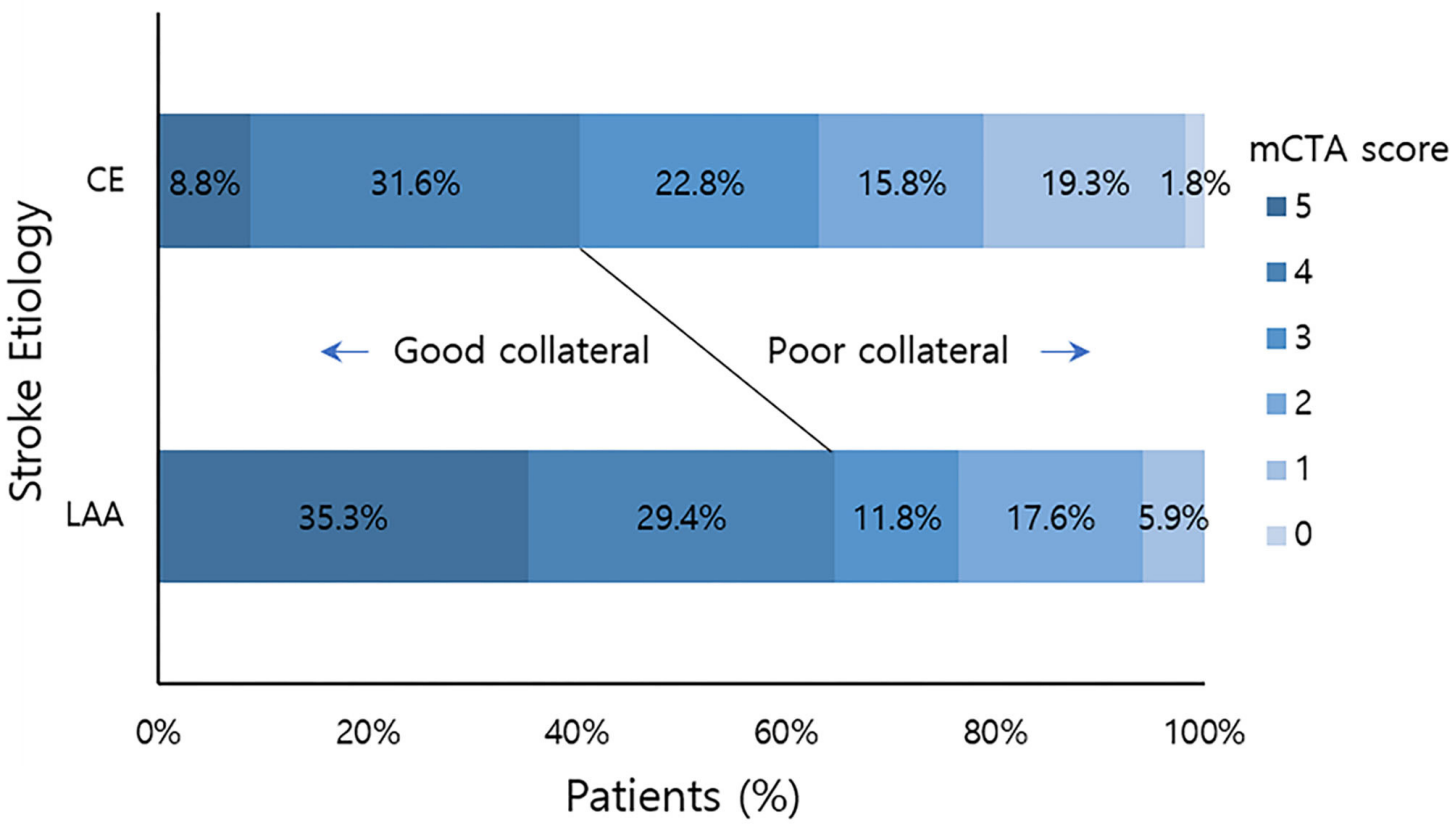
mCTA collateral flow score distribution by stroke etiology. Good collateral means mCTA collateral flow score 4–5. Poor collateral means mCTA collateral flow score 0–3. mCTA, multiphasic computed tomography angiography; LAA, large artery artherosclerosis; CE, cardio embolic.

Over the study period, 74 patients met our inclusion criteria. They were subjected to primary analysis. The mean age of these patients was 72.2 years (*SD* = 11.7 years). Of them, 28 (37.8%) were men. There were 57 (77%) patients in the CE group and 17 (23%) in the LAA group. The mean time from stroke onset to CT was 263 min (*SD* = 392 min). Their overall demographics, clinical characteristics, blood chemistry results, and mCTA scores are summarized in Table 1. Among patients with *CE*, 46 (80.7%) had atrial fibrillation. Patients with LAA were older (median: 76.4 ± 12.0 years vs. 71.4 ± 11.3 years, *p* = 0.064) with higher initial SBP (median: 170 ± 27.8 mmHg vs. 148 ± 29.1 mmHg; *p* = 0.011) than patients with CE. We found a significant shift toward better collateral flow scores in favor of stroke due to LAA (median: 3.75 ± 1.34 vs. 2.89 ± 1.33; *p* = 0.021). Furthermore, when collateral flow scores were dichotomized into good (scores of 4–5) and poor (scores of 0–3), significantly more patients with LAA had good collateral flow than those with CE [11/17 (64.7%) vs. 20/57 (40.4%)] (Figure 1).

**Table 1:** Clinical and angiographic characteristics of patients with acute ischemic stroke.

	**Total (*n =* 74)**	***CE* (*n =* 57)**	**LAA (*n =* 17)**	***p***
**Demography**
Male, *n* (%)	28 (37.8)	19 (33.3)	9 (56.2)	0.145*
Age	72.2 ± 11.7	71.4 ± 11.3	76.4 ± 12.0	0.064^†^
HTN, *n* (%)	53 (71.6)	41 (71.9)	12 (75.0)	0.856*
DM, *n* (%)	19 (25.7)	15 (26.3)	4 (25.0)	1*
Hyperlipidemia, *n* (%)	33 (44.6)	23 (40.4)	9 (56.2)	0.272*
Atrial fibrillation, *n* (%)	46 (62.2)	46 (80.7)	0 (0)	0.001 < *
Current smoking, *n* (%)	5 (6.8)	3 (5.26)	2 (12.5)	0.301*
Current alcohol consumption, *n* (%)	15 (20.3)	10 (1.75)	5 (31.2)	0.283*
Previous statin use	21 (28.4)	12 (21.1)	8 (50.0)	0.022*
Previous stroke	25 (33.8)	17 (29.8)	8 (50.0)	0.148*
**Laboratory**
SBP	153 ± 29.8	148 ± 29.1	170 ± 27.8	0.011
DBP	83.8 ± 18.6	83.5 ± 20.3	84.8 ± 11.8	0.433^†^
PR	82.9 ± 22.6	82.9 ± 24.4	83.6 ± 16.2	0.492^†^
T-chol	167 ± 45.3	169 ± 41.4	164 ± 59.4	0.431^†^
LDL	106 ± 38.4	107 ± 34.6	104 ± 50.9	0.777
HDL	52.5 ± 14.4	52.2 ± 14.5	53.3 ± 14.8	0.793
TG	130 ± 147.0	126 ± 161.0	145 ± 96.7	0.171
ProBNP	4,917 ± 10,239	5,692 ± 11,033	915 ± 1,270	0.067^†^
Creatinine	0.976 ± 0.499	1.01 ± 0.538	0.853 ± 0.335	0.212^†^
eGFR	70.9 ± 25.3	67.1 ± 22.1	84.3 ± 32.3	0.036^†^
HbA1c	6.14 ± 1.13	6.09 ± 1.16	6.44 ± 1.11	0.008^†^
Free fatty acid	1,003 ± 488	984 ± 524	1,062 ± 363	0.605
Lip(a)	21.1 ± 17.0	20.9 ± 18.6	21.7 ± 11.9	0.303^†^
**Brain imaging**
Onset to CT time (min)	263 ± 392	221 ± 282	404 ± 629	0.128^†^
mCTA score	3.08 ± 1.36	2.89 ± 1.33	3.75 ± 1.34	0.021^†^
mCTA grade				0.099^†^
Poor (0–3)	40 (54.1)	34 (59.6)	6 (35.3)	
Good (4–5)	34 (45.9)	23 (40.4)	11 (64.7)	
ASPECTS	7.96 ± 2.60	7.70 ± 2.87	8.75 ± 1.06	0.583
NIHSS score	12.3 ± 7.00	13.8 ± 6.24	7.44 ± 7.00	0.002^†^
**TTE findings**
Left atrial size, mm	42.8 ± 7.82	43.2 ± 8.41	42.3 ± 4.65	0.610
Aortic diameter, mm	34.1 ± 4.34	33.5 ± 4.28	36.4 ± 4.00	0.026
LVIDd, mm	48.6 ± 6.89	48.7 ± 7.10	48.7 ± 6.32	0.551^†^
LVIDs, mm	30.5 ± 6.96	31.0 ± 6.95	29.0 ± 6.94	0.471^†^
Left ventricular ejection fraction, %	60.3 ± 10.2	58.8 ± 10.1	65.1 ± 9.40	0.029^†^
Mitral flow E	0.953 ± 0.359	1.03 ± 0.358	0.7 ± 0.223	0.002^†^
Mitral flow e	1.19 ± 8.94	1.52 ± 10.1	0.0644 ± 0.0295	0.272^†^
Mitral flow E/e	16.3 ± 11.4	17.7 ± 12.4	11.5 ± 4.41	0.079^†^
Mitral flow A	0.821 ± 0.368	0.799 ± 0.468	0.851 ± 0.243	0.140
Mitral flow E/A	1.22 ± 0.849	1.56 ± 1.02	0.866 ± 0.405	0.040^†^
Deceleration time	234 ± 122	237 ± 137	220 ± 40.4	0.245^†^
Regional wall motion abnormality, *n* (%)	10 (13.5)	9 (15.8)	1 (6.25)	0.673*
Outcome (90 days mRS)	2.92 ± 2.04	2.98 ± 2.09	2.67 ± 2.02	0.580^†^

*SBP, systolic blood pressure; DBP, diastolic blood pressure; PR, pulse rate; T-chol, total cholesterol; LDL, low-density lipoprotein; HDL, high-density lipoprotein; TG, triglyceride; ProBNP, pro-B-type natriuretic peptide; lip(a), lipoprotein a; mCTA, multiphasic computed tomography angiography; ASPECTS, Alberta stroke program early CT score; NIHSS, National Institutes of Health Stroke Scale; TTE, transthoracic echocardiogram; LVIDd, left ventricular internal dimension at diastole; LVIDs, left ventricular internal dimension at systole; mRS, modified Rankin score*.

^*^Fisher's exact test.

*^†^Wilcoxon rank sum test*.

### Impact Factors of Collateral Flow

In logistic regression analysis, initial SBP was found to be associated with good collateral scores of 4–5 [odds ratio (*OR*): 0.995; 95% confidence interval (*CI*): 0.992–0.9979; *p* = 0.013]. However, vascular risk factors and echocardiographic parameters of systolic and diastolic dysfunction were not associated with collateral flow grade (Table 2).

**Table 2:** Logistic regression analysis for good collateral score (4–5) in all patients.

**Variables**	***OR* (95% *CI*)**	***p***
Age	0.999 (0.989–1.009)	0.838
HTN	1.116 (0.865–1.439)	0.398
DM	1.094 (0.841–1.423)	0.502
Hyperlipidemia	1.047 (0.831–1.319)	0.698
Stroke etiology	0.784 (0.600–1.025)	0.075
Atrial fibrillaton	0.745 (0.593–0.935)	0.011
Current smoking	0.938 (0.593–1.484)	0.785
Current alcohol consumption	1.099 (0.825–1.464)	0.517
Previous stroke	0.861 (0673–1.095)	0.222
Previous statin use	1.094 (0.848–1.411)	0.489
SBP	0.995 (0.992–0.999)	0.013
DBP	0.996 (0.990–1.002)	0.167
eGFR	1.002 (0.998–1.007)	0.304
HbA1c	0.970 (0.847–1.111)	0.660
Aortic diameter	0.999 (0.972–1.028)	0.960
Left ventricular ejection fraction	0.998 (0.986–1.010)	0.700
Mitral flow E	1.014 (0.712–1.444)	0.939
Mitral flow E/e	1.003 (0.992–1.014)	0.589
Mitral flow A	1.044 (0.624–1.745)	0.870
Mitral flow E/A	0.994 (0.795–1.241)	0.955
ACA A1 agenesis	0.966 (0.652–1.432)	0.086
Fetal PCA	1.009 (0.758–1.344)	0.095

*Univariable logistic analysis were built to assess the association between of independent variables and collateral flow grade. Collateral flow grade was considered as the dependent variable*.

*SBP, systolic blood pressure; DBP, diastolic blood pressure; ACA, anterior cerebral artery; PCA, posterior cerebral artery; OR, odds ratio; CI, confidential interference*.

**Figure 2:**
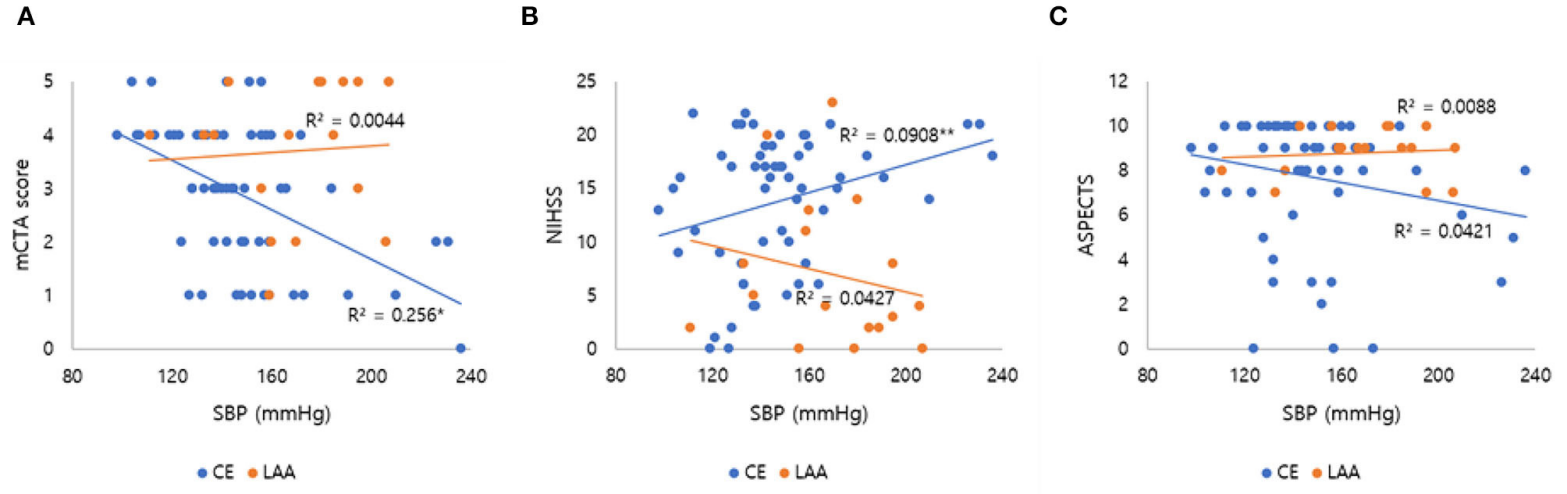
Correlation of initial SBP with mCTA score, NIHSS score, and ASPECTS. The line in each graph was obtained from the univariable linear regression. **(A)** Correlation of initial SBP and mCTA score by stroke subtypes. **(B)** Correlation of initial SBP and NIHSS score by stroke subtypes. **(C)** Correlation of initial SBP and ASPECTS by stroke subtypes. SBP, systolic blood pressure; mCTA, multiphasic computed tomography angiography; NIHSS, National institute of health stroke scale; ASPECTS, Alberta Stroke Program Early CT Score; LAA, large artery atherosclerosis; CE, cardio embolic. *p < 0.001; **p = 0.023.

**Figure 3:**
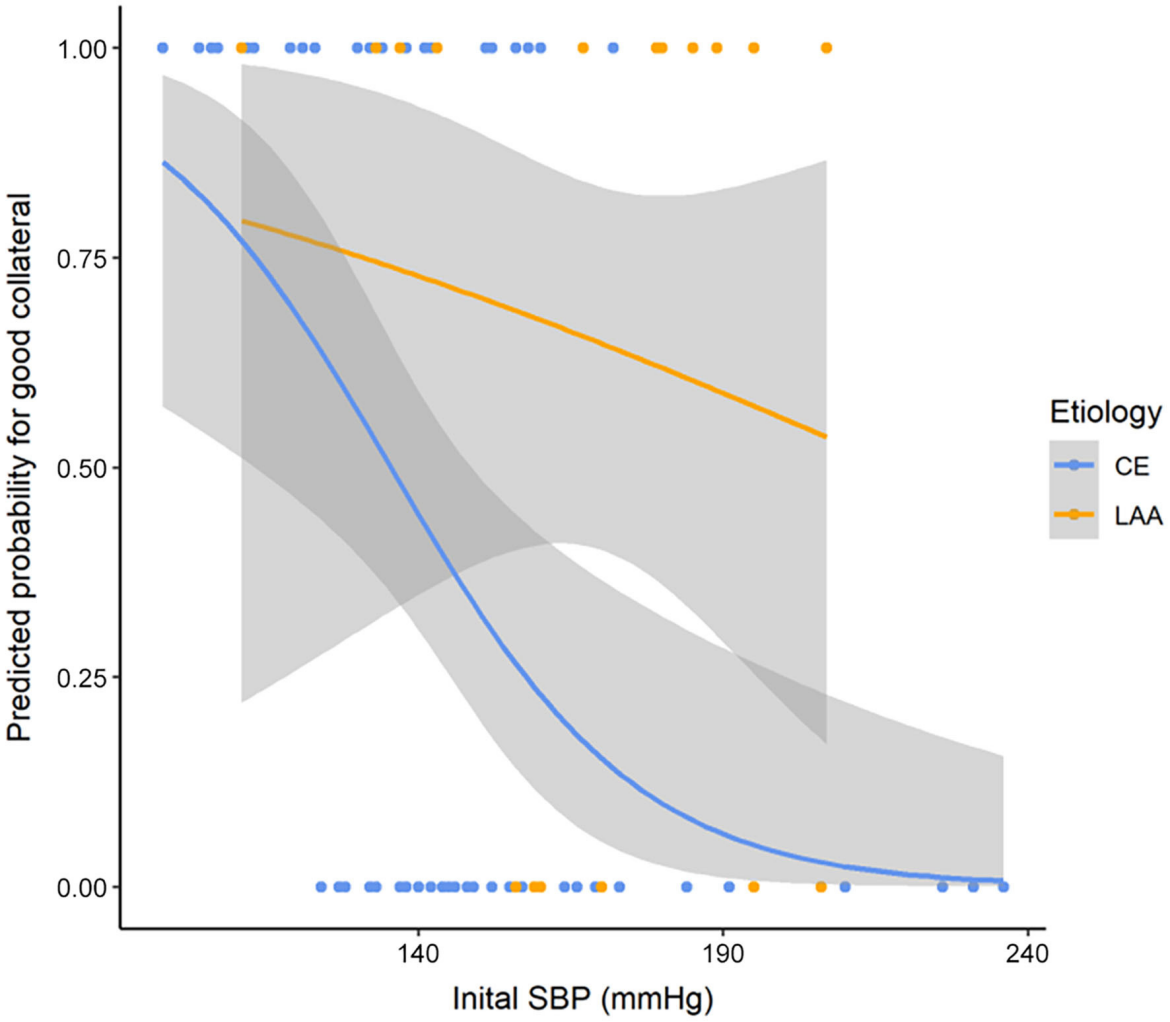
Predicted probability curve for good collateral score (4–5) and SBP by stroke etiology. *p* = 0.004 in *CE* group and *p* = 0.536 in LAA group. SBP, systolic blood pressure; LAA, large artery atherosclerosis; *CE*, cardio embolic.

After we found that initial SBP was related to collateral flow grade, we performed an appropriate multivariable logistic regression analysis to obtain OR. Stroke etiology, ACA A1 agenesis, and fetal PCA (*p* < 0.1) were chosen as covariates to further assess how different factors affected collateral flow. ASPECTS and age were further adjusted, even though these variables were not significant in the univariable analysis. Since these variables influence leptomeningeal collaterals and in general the peripheral micro-perfusion. Multivariable logistic regression analysis showed that initial SBP [odds ratio (*OR*): 0.994; 95% confidence interval (*CI*): 0.990–0.998; *p* = 0.002] and stroke etiology etiology (*OR*: 0.718; 95% *CI*: 0.548–0.940; *p* = 0.019) were independent factors of the collateral flow grade (Table 3). However, anatomical variations of circle of Willis such as agenesis of ACA A1 and fetal PCA were not associated with the collateral flow grade. In subgroup analysis, the collateral flow grade was independently associated with initial SBP in the CE group (*OR*: 0.993; 95% *CI*: 0.989–0.998; *p* = 0.004; Table 3), but not in the LAA group (*OR*: 0.992; 95% *CI*: 0.980–1.004; *p* = 0.218; Table 3). Figure 2 shows correlation initial SBP with mCTA score, NIHSS score, and ASPECTS. Initial SBP was correlated with mCTA score and NIHSS score in the CE group (*r*^2^ = 0.256, *p* < 0.001; *r*^2^ = 0.091, *p* = 0.023, respectively; Figures 2A,B). Moreover, the predicted probability of good collateral score (4–5) decreased as initial SBP increased in CE group (*p* = 0.004) but not in LAA group (*p* = 0.536; Figure 3). The probability of having good collateral is ~50% at SBP of 140 mmHg. Supplementary Figure 3 shows the correlation of NIHSS score, ASPECT score, and mCTA score. In CE group, ASPECT score and mCTA score were well correlated (*r*^2^ = 0.151, *p* = 0.003; Supplementary Figure 3D).

**Table 3:** Multivariable logistic regression analysis for good collateral score (4–5) in all patients and subgroup analysis.

	**All patients**		**CE**		**LAA**	
	***OR* (95% *CI*)**	***p***	***OR* (95% *CI*)**	***p***	**OR (95% *CI*)**	***p***
Stroke etiology	0.718 (0.548–0.940)	0.019				
Age	1.003 (0.991–1.010)	0.950	0.996 (0.985–1.006)	0.422	1.014 (0.992–1.037)	0.248
SBP	0.994 (0.990–0.998)	0.002	0.993 (0.989–0.998)	0.004	0.992 (0.980–1.004)	0.218
ACA A1 agenesis	1.116 (0.771–1.617)	0.562	1.059 (0.713–1.571)	0.778	1.323 (0.444–3.947)	0.626
Fetal PCA	0.860 (0.653–1.132)	0.285	0.870 (0.645–1.174)	0.366	0.731 (0.324–1.647)	0.465
ASPECT	1.043 (0.999–1.089)	0.061	1.037 (0.993–1.083)	0.110	1.124 (0.884–1.428)	0.361

*Multivariable logistic analysis were built to assess the association between of initial SBP and collateral flow grade. In multivariable analysis, further adjustment on etiology, age, SBP, ACA A1 agenesis fetal PCA and ASPECT. In subgroup analysis, further adjustment on SBP, ACA A1 agenesis and fetal PCA*.

*SBP, systolic blood pressure; ACA, anterior cerebral artery; PCA, posterior cerebral artery;ASPECTS, Alberta stroke program early CT score; OR, odds ratio; CI, confidential interference; CE, cardiac embolic; LAA, large artery atherosclerosis*.

## Discussion

In this study, we identified that elevated initial SBP was associated with poor cerebral collateral flow and more severe symptoms in the CE group. In the contrary, these associations with SBP were not observed in the LAA group. To the best of our knowledge, these results are novel findings. With the correlation analysis, initial SBP elevation was associated with poor mCTA score and higher NIHSS score in the CE group. According to our regression results, poor collateral flow can be predicted with initial SBP in the CE group, but not in the LAA group. Moreover, poor collateral flow was associated with lower ASPECT score in the CE group. This means that collateral flow is more important for predicting the clinical manifestation and ischemic tissue damage of the CE group. However, the number of patients is too small, so conclusion should be cautious especially in the LAA group.

The patients with AIS-LVO due to LAA had a better collateral flow than CE stroke. The association of stroke subtype and collateral flow has been reported by previous studies (6–8).

Previous studies have also reported elevated mean SBP levels in patients with AIS-LVO showing that patients with LAA had higher initial SBP than patients with CE (10, 16). Kim et al. have found that patients with non-CE have higher baseline SBP than patients with embolic stroke (17). The sustained better collateral flow with blood pressure elevation in the LAA group might be explained by the gradual development of collaterals. Primary collaterals refer to arterial segments of the circle of Willis. In the event of large intracranial artery occlusions, these primary collaterals can provide immediate diversion of cerebral blood flow (CBF) to ischemic regions through existing anastomoses. Secondary collaterals include the ophthalmic artery and leptomeningeal arteries as well as other anastomoses between the distal, small-caliber arteries. Tertiary collaterals refer to newly developed micro vessels through angiogenesis at the periphery of ischemic regions. Meanwhile, secondary collaterals might be anatomically present, although up regulating the capacity of these alternative routes to support CBF might require time (18). Although angiogenesis, sprouting of new capillaries from postcapillary venules, is active during chronic hypoxia (19), arteriogenesis is triggered by fluid shear stress rather than by hypoxia. The growth of cerebral collaterals is correlated with the rising intravascular flow rate. Thus, elevated SBP may increase the intravascular flow rate, which in turn facilitates the development of cerebral collaterals through arteriogenesis. Patients with LAA might have adapted to elevated SBP due to chronic hypoperfusion, resulting in a good collateral flow. Previous studies have found that lower SBP is associated with less hemodynamic improvement during follow-up in patients with atherosclerotic internal carotid artery (ICA) or MCA disease (20). In patients presenting with AIS-LVO with proximal MCA occlusion, initial recruitment of collaterals occurs rapidly (21). But in the CE cases, the event of a large artery occlusion is abrupt and there is no time for the development of tertiary collaterals. Therefore, a limited recruitment of collateral channels and ongoing cerebral edema may cause blood pressure elevation in the CE group to maintain cerebral perfusion pressure. But higher blood pressure can also facilitate further tissue damage through cerebral edema and hemorrhagic transformation (22, 23). In case of futile recanalization after endovascular thrombectomy, cerebral edema volume is increased by same mechanism (24).

Wufuer et al. have reported that lower SBP is associated with good collateral flow in patients with ICA occlusion (11). But our study found no relationship between collateral flow grade in stroke patients with LAA and initial SBP. The inverse association between poor collateral flow and high SBP in the CE group was not prominent in the LAA group. This is because they already have elevated SBP. However, whether the variability of blood pressure affects the collateral flow remains unclear.

In this study, there were differences in the baseline premorbid statin use, HbA1c, eGFR, and echocardiographic parameters between the 2 groups. However, these findings were not associated with the collateral flow grade in multivariable regression analysis. Lee et al. have reported that premorbid use of statin in atrial fibrillation patients is associated with an excellent collateral flow (25). Unlike the previous report, there was no association between collateral flow and premorbid use of statin in our study. This might be due to differences in the baseline characteristics of enrolled patients. They included MCA M1 or M2 occlusion and excluded AIS-LVO due to LAA. Anatomical variations of circle of Willis such as agenesis of ACA and fetal PCA were not associated with the collateral flow grade in our study. A good collateral flow is more frequently associated with the development of collaterals in the anterior communicating artery, the posterior communicating artery, and the leptomeningeal collateral (11). Up to now, there is no confirmative conclusion regarding the effect of collaterals *via* the circle of Willis (26).

The strength of our study is that included patients were exclusively M1 total occlusion with mCTA identified within 24 h (263 ± 392 min), which can analyze the collateral flow in a strictly controlled environment. Second, all the echocardiographic parameters were investigated which might affect cardiac output and cerebral blood flow. Third, as for the strategy of acute stroke treatment, out data may have clinical implication on acute stroke management. Lowering blood pressure in LAA may disturb physiologic compensation of cerebral collateral flow especially at higher SBP range. In the contrary, CE stroke with high initial SBP has less possibility of having good collateral flow. Therefore, it is reasonable to perform early and rapid recanalization during hyperacute period and monitor the development cerebral edema during later period in these patients.

Our study has several limitations, mostly inherent to its retrospective design and relatively small sample size in a single center. Multicenter trials and larger-scale longitudinal study are warranted to examine whether blood pressure contributes to collateral flow. Second, this study examining blood pressure and collateral flow was cross-sectional, thus allowing us to detect associations without formulating a causal link. Third, the majority of patients with CE stroke etiology in our study had atrial fibrillation. Atrial fibrillation might be relatively more prevalent than other CE sources in patients with LVO. However, other major CE stroke etiology can also cause abrupt vessel occlusion, which is basically different from the mechanism of LAA. Lastly, although it is generally considered that digital subtraction angiography (DSA) is the golden standard for collateral evaluation. mCTA is a quick, easy and non-invasive method associated with clinical outcome, which are merits over DSA (27).

## Conclusion

In conclusion, poor cerebral collateral flow in AIS-LVO was associated with elevated initial SBP. The association of collateral flow with initial SBP was significant in CE group, but not in LAA group. However, the number of patients is too small, so conclusion should be cautious especially in LAA group. Further study is needed to understand the mechanisms related to differential effects of SBP on cerebral collateral flow by stroke subtypes.

## Data Availability Statement

The raw data supporting the conclusions of this article will be made available by the authors, without undue reservation.

## Ethics Statement

The studies involving human participants were reviewed and approved by the Ethical Committee of the Samsung Seoul Medical Center. The patients/participants provided their written informed consent to participate in this study.

## Author Contributions

JS established the study concept, analyzed and interpreted the data, and wrote the manuscript. J-WC, W-KS, and OB established study database and made critical revision of the manuscript. G-MK established the study idea and database and made critical revision of the manuscript with intellectual contents. All authors contributed to the article and approved the submitted version.

## Conflict of Interest

The authors declare that the research was conducted in the absence of any commercial or financial relationships that could be construed as a potential conflict of interest.

## Publisher's Note

All claims expressed in this article are solely those of the authors and do not necessarily represent those of their affiliated organizations, or those of the publisher, the editors and the reviewers. Any product that may be evaluated in this article, or claim that may be made by its manufacturer, is not guaranteed or endorsed by the publisher.
