# Supplementary material for: Association of Systolic Blood Pressure and Cerebral Collateral Flow in Acute Ischemic Stroke by Stroke Subtype
Source: Front Neurol. 2022 May 13;13:863483. doi: 10.3389/fneur.2022.863483 (PMC9136006; doi:10.3389/fneur.2022.863483)

Supplementary figure 1. Flow diagram of patient selection

mCTA; multiphasic computed tomography angiography, MCA; middle cerebral artery, LAA; large artery atherosclerosis, CE; cardio embolic

Supplementary figure 2. The example of mCTA score

A score on a scale of 0 to 5 was given, with 0 being the worst and 5 being the best: (**0**, no vessels visible in the affected hemisphere in any phase; **1**, only a few vessels were visible in the affected hemisphere in any phase; **2,** a filling delay of two phases in the affected hemisphere with a significantly reduced number of vessels in the ischemic territory, or one phase delay showing regions without visible vessels; **3,** a filling delay of two phases in the affected hemisphere, or a delay of one phase with a significantly reduced number of vessels in the ischemic territory; **4,** a filling delay of one phase in the affected hemisphere, but the extent and prominence of pial vessels were the same; **5,** no filling delay compared to the asymptomatic contralateral hemisphere, with normal pial vessels in the affected hemisphere).

Supplementary figure 3. Correlation of mCTA score with NIHSS score and ASPECTS

The line in each graph was obtained from the univariable linear regression. R means correlation coefficients. (A) Correlation of NIHSS score and mCTA score in all patients. (B) Correlation of ASPECTS and mCTA score in all patients. (C) Correlation of NIHSS score and mCTA score by stroke etiology. (D) Correlation of ASPECTS and mCTA score by stroke etiology.

mCTA, multiphasic computed tomography angiography; ASPECTS, Alberta stroke program early CT score; NIHSS, National Institutes of Health Stroke Scale

Supplementary figure 1. Patients enroll tree

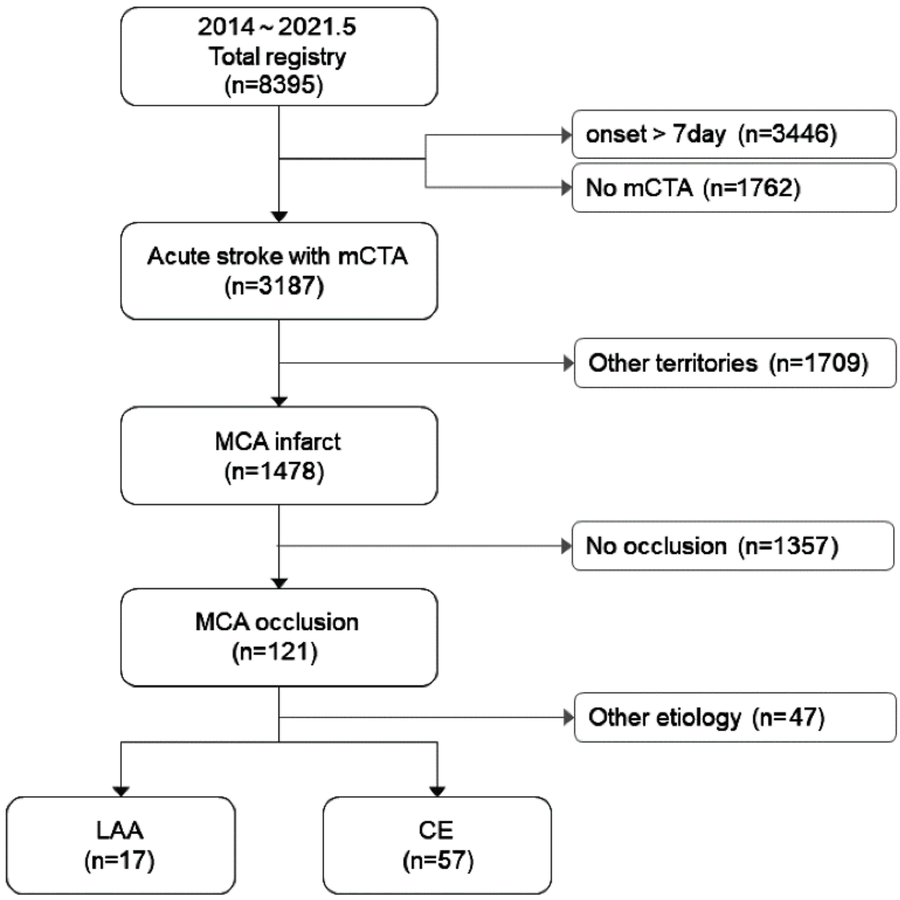

Supplementary figure 2. The example of mCTA score

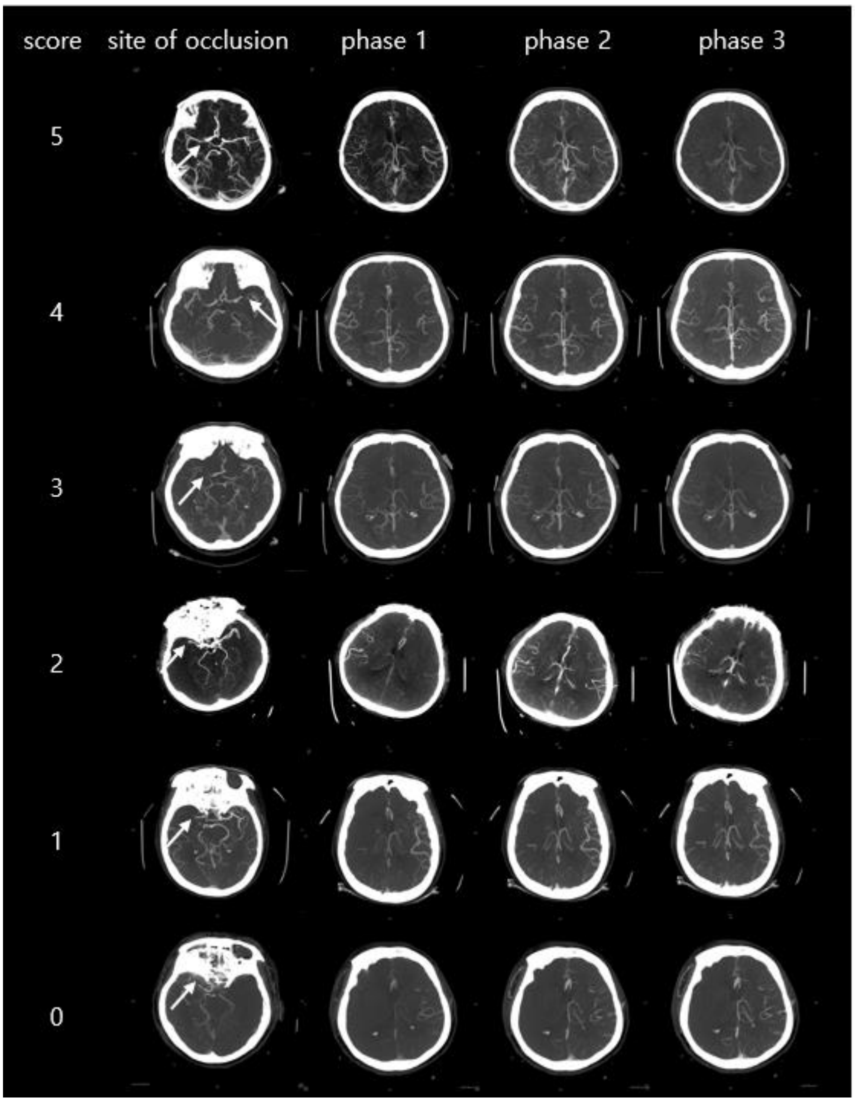

Supplementary figure 3. Correlation of mCTA score with NIHSS score and ASPECTS

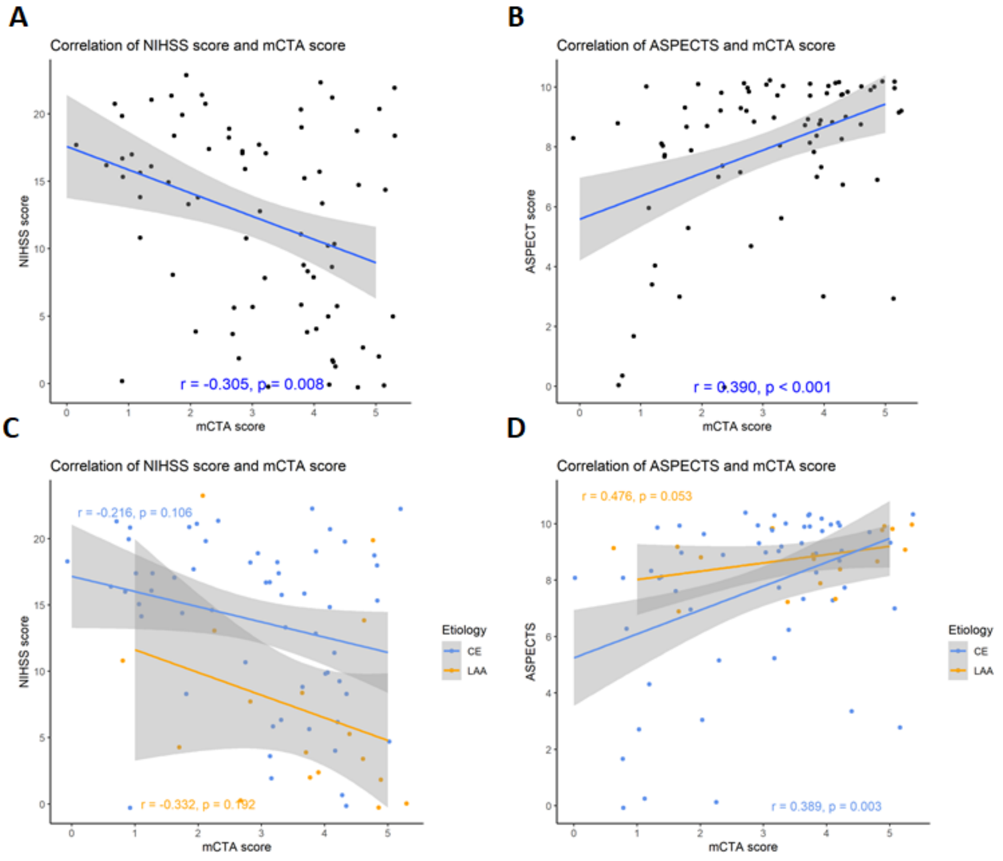

Supplement: Supplementary file 1 [file Data_Sheet_1.pdf]
